# Analog Approach to Constraint Satisfaction Enabled by Spin Orbit Torque Magnetic Tunnel Junctions

**DOI:** 10.1038/s41598-018-24877-z

**Published:** 2018-05-02

**Authors:** Parami Wijesinghe, Chamika Liyanagedera, Kaushik Roy

**Affiliations:** Purdue University, School of Electrical and Computer Engineering, West Lafayette, Indiana, 47907 USA

## Abstract

Boolean satisfiability (*k*-SAT) is an NP-complete (k ≥ 3) problem that constitute one of the hardest classes of constraint satisfaction problems. In this work, we provide a proof of concept hardware based analog k-SAT solver, that is built using Magnetic Tunnel Junctions (MTJs). The inherent physics of MTJs, enhanced by device level modifications, is harnessed here to emulate the intricate dynamics of an analog satisfiability (SAT) solver. In the presence of thermal noise, the MTJ based system can successfully solve Boolean satisfiability problems. Most importantly, our results exhibit that, the proposed MTJ based hardware SAT solver is capable of finding a solution to a significant fraction (at least 85%) of hard 3-SAT problems, within a time that has a polynomial relationship with the number of variables(<50).

## Introduction

The Boolean satisfiability problem investigates whether there exists an assignment for the input variables that satisfies a given Boolean formula. *k*-SAT is widely used in many practical applications including automated planning^1^, test pattern generation^2^, hardware model checking^3^, software program testing^4^ and timing analysis^5^. *k*-SAT problems are NP-complete (*k* ≥ 3)^6,7^. *i*.*e*., there are no known algorithms that can guarantee a solution for a SAT problem in polynomial time, making it extremely difficult to solve most satisfiability problems with reasonable computational resources. Numerous research efforts have been directed towards realizing improved SAT solvers^8–13^, since a polynomial time solution to *k*-SAT implies efficient solutions to a large number of hard optimization problems. The standard conjunctive normal form (CNF) of any Boolean *k*-SAT problem with *N* variables can be written as

1 $$ {\mathcal F} =({x}_{1}\cup {x}_{2}\cup {\overline{x}}_{3})\cap ({x}_{2}\cup {\overline{x}}_{1}\cup {x}_{5})\cap \mathrm{...}\,({\overline{x}}_{4}\cup {\overline{x}}_{5}\cup {x}_{3})$$

where *x*_*i*_∈{0, 1} is a variable and each clause is the disjunction (OR, ∪) of *k* (*k* = 3 in this case) such variables or their negation (${\bar{x}}_{i}$). The propositional formula ${\mathcal F}$ is a conjunction (AND, ∩) of *M* number of such clauses. The hardness of a SAT problem can be measured as the ratio between the number of clauses and the variables, known as the constraint density *α*_*c*_ (Supplementary section S1).

Analog computational approaches have recently demonstrated promising results in a diverse array of applications^14^ including aforementioned constraint satisfaction^8,9,15^. A recent analog formulation of a *k*-SAT solver has demonstrated its potential on locating a solution for the Boolean satisfiability problem in polynomial continuous-time^8^. However, implementing this set of analog formulae using a digital computer will diminish the polynomial time benefits, due to varying computational complexities between different time steps. Also, a hardware implementation of this analog *k*-SAT solver^8^ is not ideal^16^, due to the exponential energy fluctuations in the system. Consequently, a Cellular Neural Network (CNN) based analog SAT solver with bounded variables was proposed^9^, and it is more appealing for hardware implementations. Although this bounded system does not have polynomial time complexity, noise effects in the analog hardware can potentially reduce the long transient times^9^ in the system, as we demonstrate in this work. The dynamics of this analog SAT solver, which is also the framework of our work, can be defined by the following set of equations^9^.

2 $${\dot{s}}_{i}(t)=\frac{d{s}_{i}(t)}{dt}=-\,{s}_{i}(t)+Af({s}_{i}(t))+\sum _{m}{c}_{mi}\,g({a}_{m}(t))$$

3 $${\dot{a}}_{m}(t)=\frac{d{a}_{m}(t)}{dt}=-{a}_{m}(t)+Bg({a}_{m}(t))-\sum _{i}{c}_{mi}\,f({s}_{i}(t))+1-k$$

Here the variable *s*_*i*_ represents the state of the *i*^*th*^ (*i* = 1, 2, ..., *N*) Boolean variable (*x*_*i*_) and *a*_*m*_ represents the “satisfiedness” of the *m*^*th*^ (*m* = 1, 2, ..., *M*) clause of the Boolean function. *C* is the problem specific ‘interconnection matrix’ of size *M* × *N* (Supplementary section S1). The functions *f*() and *g*() are the thresholding functions applied on variables *s*_*i*_ and *a*_*m*_, respectively, as follows

4 $$f({s}_{i})=\frac{1}{2}(|{s}_{i}+\mathrm{1|}-|{s}_{i}-\mathrm{1|})$$

5 $$g({a}_{m})=\frac{1}{2}(1+|{a}_{m}|-\mathrm{|1}-{a}_{m}|)$$

This system is explained in detail in the Supplementary section S1. It is mathematically shown^9^ that these set of equations satisfy three theorems that demonstrate the properties of the model. The same theorems are used in Supplementary section S4, to show that our hardware SAT solver demonstrates the same properties. Following are the three theorems.

- Theorem 1: Variables *s* and *a* remain bounded.
- Theorem 2: Every *k*-SAT solution has a corresponding stable fixed point.
- Theorem 3: A stable fixed point always corresponds to a solution.

We present a hardware platform built on nano-scale spintronic devices, which can successfully emulate the behaviour of the aforementioned SAT solver. As a matter of fact, recent studies have demonstrated efficient hardware models that utilize the underlying device physics of nano-electronic structures to perform computationally intensive calculations^17–21^. In this work, each of the above differential equations is modeled by a single MTJ with an underlying heavy metal (*Ta*, *Pt*, etc.) layer. Our numerical results demonstrate that, the MTJ based SAT solver exhibits a polynomial dependency, between the number of variables and the real time for convergence, even for SAT problems that are known to be hardest to solve (note that this is not a mathematical proof that shows a guaranteed polynomial time complexity). We conjecture that, this is due to the non-deterministic nature of our system caused by the random thermal noise, and also due to the added complexities associated with MTJs.

### Using the behaviour of Magnetic Tunnel Junctions for a SAT solver

**Figure 1:**
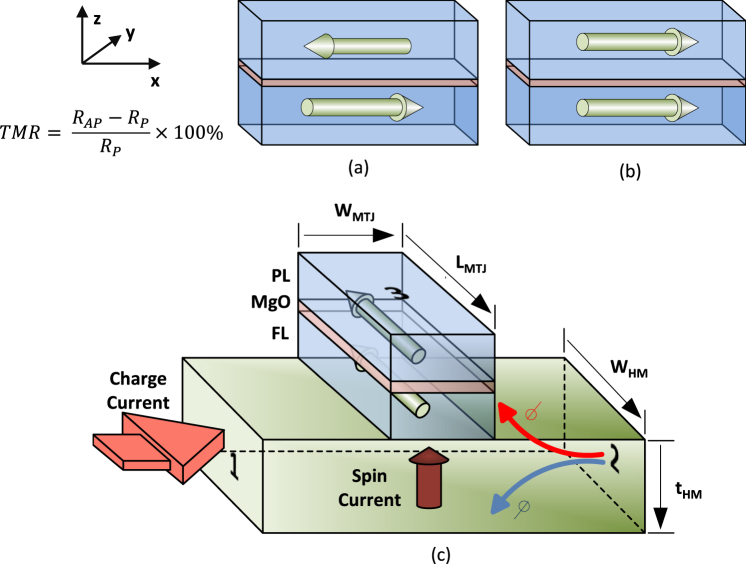
The Heavy Metal-Magnetic Tunnel Junction structure. (**a**) High resistive (*R*_*AP*_) anti-parallel state of an MTJ (**b**) Low resistive (*R*_*P*_) parallel state of an MTJ. The Tunnel Magneto-Resistance (TMR) is a measure of the normalized difference of these resistances. Typical values of the TMR ranges from 150%–600%^41,42^. (**c**) An HM-MTJ structure. The charge current through the HM layer underneath the MTJ, gets split into up and down spins, inducing a perpendicular spin current which can reverse the magnetization of the free layer through the Spin Orbit Torque phenomenon.

The proposed hardware based SAT-solver is a collection of heavy metal-MTJ (HM-MTJ) structures, interfaced through simple CMOS peripheral circuitry as described in the next section. The device-circuit structure we propose is generic and can be adapted to solve a given k-SAT problem. The HM-MTJ structure is composed of two ferromagnetic layers called the Pinned Layer (PL) and the Free Layer (FL), separated by a thin tunneling oxide (*MgO*) layer and an HM under-layer (Fig. 1(c)). The PL magnetization direction ($\hat{p}$) is fixed and acts as a reference. In contrast, the magnetization direction $\hat{m}$ of the FL can be switched by passing a current through the HM under-layer, using the Spin Orbit Torque (SOT) phenomenon. Such a technique has emerged as an energy-efficient mechanism for magnetization reversal^22–24^. Furthermore, the three terminal structure of this MTJ with a heavy metal under layer is beneficial in this work due to the possibility of simultaneous read and write to an MTJ^25^. This is impossible to achieve in a two terminal MTJ structure that requires the write current to flow through the tunnel junction.

The resistance measured across the MTJ varies with the magnetization of the FL, and shows two stable states; high resistive (*R*_*AP*_) anti-parallel (AP) state, and low resistive (*R*_*P*_) parallel (P) state. Equations 6 and 7 depict how the resistance across the MTJ (*R*_*MTJ*_) varies with the direction of the FL magnetization, where *θ*_*fp*_ is the angle between the directions of FL and PL magnetizations^26^. The magnetization reversal dynamics of an MTJ with an applied current is explained in the Supplementary section S2 using the Landau-Lifshitz-Gilbert-Slonczewski (LLGS) equations^27^. The speed of this magnetization reversal can be controlled by the magnitude of the current passing through the HM layer. However, due to the effect of random thermal noise on nano-scale magnets, the MTJ switching speed follows a Gaussian distribution.

6 $${R}_{MTJ}={(\frac{1}{{R}_{P}}{(cos(\frac{{\theta }_{fp}}{2}))}^{2}+\frac{1}{{R}_{AP}}{(sin(\frac{{\theta }_{fp}}{2}))}^{2})}^{-1}$$

7 $${\theta }_{fp}=co{s}^{-1}(\hat{m}\,.\,\hat{p})$$

The resistance across an MTJ, *R*_*MTJ*_, can be easily converted into a voltage by using a simple resistor divider circuit. In the proposed hardware implementation of the SAT solver, each *s*_*i*_ and *a*_*m*_ variable from equations 2 and 3 are represented using a single HM-MTJ structure. The state of these variables at a particular time instant are given by the resistance across the corresponding MTJ device. The couplings between the *s* and *a* variables (terms ${\sum }_{m}{c}_{mi}\,g({a}_{m}(t))$ and $-\,{\sum }_{{\rm{i}}}{c}_{mi}\,f({s}_{i}(t))$ in equations 2 and 3) are mapped as currents through the HM layer using the interface circuitry explained in the next section.

**Figure 2:**
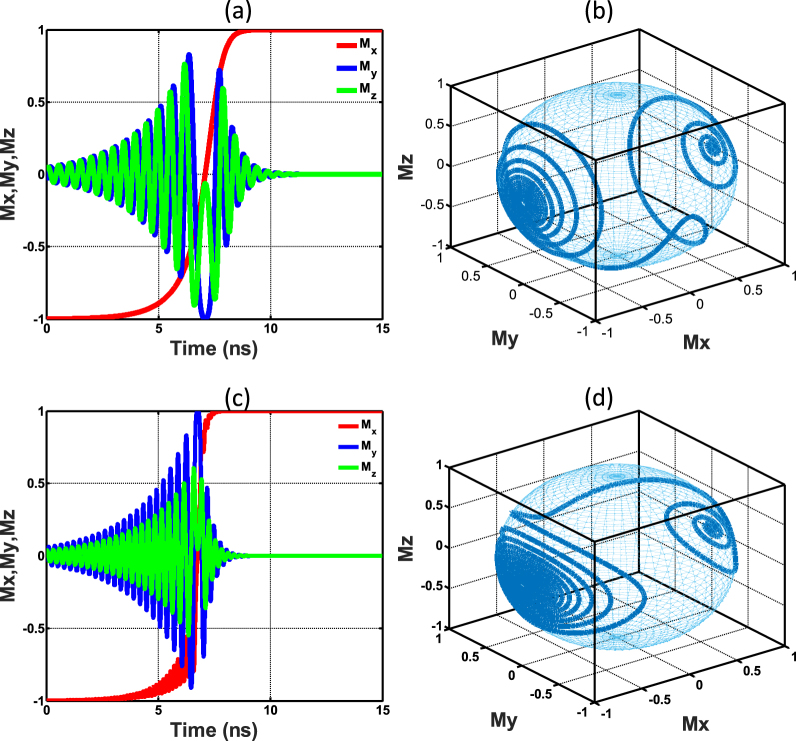
An MTJ changing its state from P to AP due to an applied current. (**a**) Time evolution of the components of unit magnetization vector in an MTJ with a FL thickness of *t*_*ss*_ (**b**) Unit magnetization traversal (in 3 dimensional space) of an MTJ with a FL thickness of *t*_*ss*_ (**c**) Time evolution of the components of unit magnetization vector in an MTJ with a FL thickness larger than *t*_*ss*_ (**d**) Unit magnetization traversal (in 3 dimensional space) of an MTJ with a FL thickness larger than *t*_*ss*_. Notice the lack of oscillations in *M*_*x*_ in (**a**), during the magnetization reversal of the FL, in contrast to (**c**).

In addition to the mathematical explanation that can be found in Supplementary section S4, we now intuitively explain how our MTJ based SAT solver mimics the system elaborated in equations (2–3). One main feature of this system is that, the current values of the variables depend on their previous states as well as some inputs. Similarly, the FL magnetization of an MTJ depends on its previous magnetization as well as the driving current. Another feature of the system in (2-3) is that, when the feedback from *a*_*m*_ towards the dynamics of *s*_*i*_ (*i*.*e*., ${\sum }_{m}{c}_{mi}\,g({a}_{m}({t}_{0}))$) is zero after a particular time *t*_0_, *s*_*i*_ will move towards +*A* if *s*_*i*_(*t*_0_) > 0, and −*A* if *s*_*i*_(*t*_0_) < 0, provided *A* > 1. Similarly, in an MTJ, an instantaneous removal of a current through the HM layer, will lead the free layer magnetization to settle down either to the parallel state or to the anti-parallel state. The state to which the magnet settles down is highly dependent upon the resistance it had at the time of removal of the current, in the absence of thermal noise. When the angle between the PL and FL magnetization directions ${\theta }_{fp} > \frac{\pi }{2}$ $({\theta }_{fp} < \frac{\pi }{2})$, and if the drive current is zero, then the final FL magnetization will settle down to the anti-parallel (parallel) state. However, it should be noted that this phenomenon occurs under certain conditions. In this work, we optimized the FL thickness according to the following equation to exhibit the above behaviour.

8 $${t}_{ss}=\frac{2{K}_{i}}{({N}_{zz}-{N}_{yy}){\mu }_{0}{M}_{s}^{2}}$$

where *K*_*i*_ is the energy density constant for interface perpendicular anisotropy and *N*_*zz*_, *N*_*yy*_ are the demagnetization factors along *y* and *z* directions. The derivation of this FL thickness is explained in detail in the supplementary documentation (section S3). The new traversal of the magnetization of a device with a thickness of *t*_*ss*_ is illustrated in Fig. 2(a),(b). Figure 2(c),(d) depicts the magnetization traversal of an MTJ with a thickness larger than *t*_*ss*_ for reference. Note the smooth transition of the FL magnetization component along the easy axis (denoted as *M*_*x*_) in Fig. 2(a) in contrast to the oscillatory transition of that in Fig. 2(c). In the light of this observation, we name *t*_*ss*_ as the seamless switching thickness of an MTJ.

### Structure of the SAT solver

**Figure 3:**
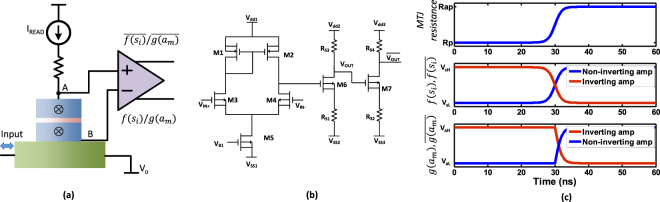
(**a**) The MTJ circuit for an *s*/*a* variable. The input current through the HM layer will change the state of the MTJ and this change will be measured by the amplifiers. The read current is assumed to be constant and its magnitude should be small so that it does not hinder the proper operation of the system. The MTJ resistance changes with the input current. The non-inverting output generates the ‘state’ (*f*(*s*_*i*_)/*g*(*a*_*m*_)) and the inverting output generates the ‘inverse-state’ ($\overline{f({s}_{i})}/\overline{g({a}_{m})}$) of an MTJ. (**b**) The reference circuit of the differential amplifier. *V*_*OUT*_ is the non-inverting output and $\bar{<mml:mpadded xmlns:xlink="http://www.w3.org/1999/xlink" voffset="0">{V}_{OUT}</mml:mpadded>}$ is the inverting output. (**c**) The outputs (*f*(*s*_*i*_)/*g*(*a*_*m*_)), varying with the MTJ resistance from *R*_*P*_ to *R*_*AP*_.

**Figure 4:**
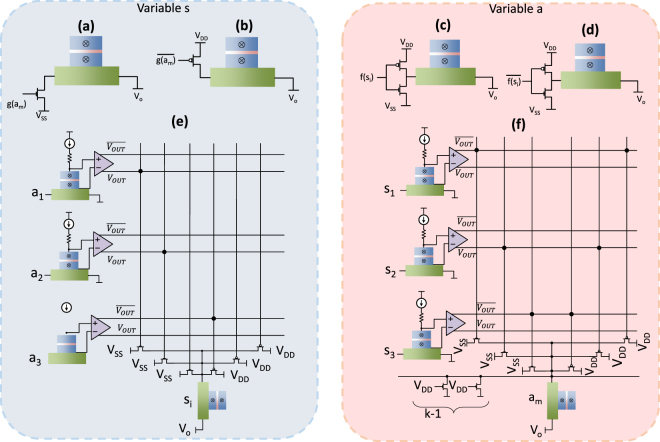
The input connection diagram of the SAT solver. The input connection to an *s*_*i*_ node from *a*_*m*_, if (**a**) *c*_*mi*_ is negative (**b**) *c*_*mi*_ is positive. The input connection to an *a*_*m*_ node from *s*_*i*_, if (**c**) *c*_*mi*_ is positive (**d**) *c*_*mi*_ is negative. Here the charge current from left to right through the HM layer drives the MTJ towards the AP state. The value of *V*_0_ is smaller than *V*_*DD*_ but larger than *V*_*SS*_. The sizing of the transistors must be done appropriately. (**e**) Outputs of three *a* nodes connected to an *s*_*i*_ node. The connection parameters (*c*_*1i*_, *c*_2i_ and *c*_3i_) between *s*_*i*_ and *a*_1_, *a*_2_ and *a*_3_ are −1, −1 and 1, respectively. (**f**) Outputs of three *s* nodes connected to an *a*_*m*_ node. The connection parameters (*c*_*m*1_, *c*_*m*2_ and *c*_*m*3_) between *a*_*m*_ and *s*_1_, *s*_2_ and *s*_3_ are −1, 1 and −1, respectively.

In this section, we will elaborate how we mapped the system in (2-3) to an array of HM-MTJs with a CMOS control interface. Each *s* and *a* variable in (2-3) is represented by the resistance of an MTJ. The resistance of the MTJ can be read as a voltage difference, when a constant read current (*I*_*READ*_) flows through the MTJ. Note that this read current must be sufficiently small (<1*μA*) to not to interfere with the proper operation of the system. The functions *f*() and *g*() can be generated efficiently using a differential amplifier shown in Fig. 3(a). Note that the amplifier is connected to the bottom of the magnet (not the heavy metal layer). This is to avoid the small variable voltage (Δ*V*) induced across the HM layer due to the varying current that flows through it. These amplifiers will increase the voltage differences incurred due to the changing resistance of the MTJs. The state AP results in a larger voltage difference between nodes *A* and *B* with respect to that resulting from P state. In our structure, the AP and the P states of an MTJ that represents an *s* variable, gets mapped in to +1 and −1 states in equation (2) respectively. In a *k*-SAT problem, a variable can appear as *x*_*i*_, or its negation (${\bar{x}}_{i}$) in the *m*^*th*^ clause. This information is encoded in the elements of the connection matrix *c*_*mi*_, as explained in Supplementary section S1. In order to account for different values of *c*_*mi*_ at the circuit level, we generate the ‘state’ (*f*(*s*_*i*_)/*g*(*a*_*m*_)) and the ‘inverse-state’ ($\overline{f({s}_{i})}/\overline{g({a}_{m})}$) signals of an MTJ. These signals are produced at the amplification stage outputs, as shown in Fig. 3(b). A differential amplifier is employed to read the voltage difference across an MTJ. Additionally, a source degenerated common source amplifier is used as a second amplification stage, to boost the voltage to the desired levels. A third amplifier is employed in the design to generate the aforementioned inverse functions ($\overline{f()}$ and $\overline{g()}$). The complete schematic of the amplification stages used in this work is shown in Fig. 3(b). The same amplifier architecture with different control voltages was used for interfacing with MTJs representing both *a* and *s* variables. The outputs *V*_*OUT *_ (*f*(*s*_*i*_) or *g*(*a*_*m*_)) and $\bar{<mml:mpadded xmlns:xlink="http://www.w3.org/1999/xlink" voffset="0">{V}_{OUT}</mml:mpadded>}$ $\overline{(f({s}_{i})}$ or $\overline{g({a}_{m}))}$ are used to drive the MOSFETs controlling the current through the heavy metal layers (Fig. 4).

Figure 3 (c) elaborates how the above mentioned ‘states’ and ‘inverse states’ vary with the resistance of an MTJ. Each differential amplifier output will vary between a predefined high voltage (*V*_*sH*_, *V*_*aH*_), and a low voltage (*V*_*sL*_,*V*_*aL*_). Therefore, the state −1 and +1 of variable *s* will be mapped to *V*_*sL*_ and *V*_*sH*_ at the non-inverting (*V*_*sH*_ and *V*_*sL*_ at the inverting) output. For the variable *a*, the non-inverting (inverting) output will be *V*_*aL*_ (*V*_*aH*_) when the resistance of the MTJ is less than (*R*_*ap*_ + *R*_*p*_)/2 and *V*_*aH*_ (*V*_*aL*_) when the resistance is *R*_*ap*_.

The term ${\sum }_{m}{c}_{mi}\,g({a}_{m}(t))$ in equation 2, and the term $-{\sum }_{i}{c}_{mi}\,f({s}_{i}(t))+1-k$ in equation 3 (the coupling between variable *s*_*i*_ and *a*_*m*_) are mapped as currents through the HM layers of MTJs, that represent *s* variables and *a* variables, respectively. At a particular time instant when the *m*^*th*^ clause is not satisfied, if the connection parameter *c*_*mi*_ is positive, the current should drive the MTJ that represents variable *s*_*i*_ towards the AP state. Similarly, when *c*_*mi*_ is negative, the current should drive that MTJ towards the P state, and when *c*_*mi*_ is zero, the current through the HM should be zero. Figure 4(a,b) graphically explains how this is realized at the circuit level. Figure 4(c,d) shows the circuit realization of the feedback from the *s*_*i*_ variable acting on the *a*_*m*_ variable, depending upon the connection parameter *c*_*mi*_. When *c*_*mi*_ is positive (negative), −*c*_*mi*_ *f*(*s*_*i*_(*t*)) should drive the MTJ that represents *a*_*m*_ towards the P (AP) state (for a case where *s*_*i*_ is in AP state). The two transistor structures (heavy metal current controllers) in Fig. 4(c,d) should provide an output voltage of *V*_*o*_, when the input $f({s}_{i}(t))=\overline{f({s}_{i}(t))}=({V}_{DD}+{V}_{SS})/2$. This is to make sure that there is no current through the HM layer when *s*_*i*_(*t*) = 0. For the Fig. 4, we assume that a charge current from left to right through an HM layer, drives the MTJ on top, towards the AP state. Figure 4(e,f) illustrates the final structure of the SAT solver with all the control logic. The connections in the ‘network’ depends on the SAT problem to be solved. Therefore, the connecting switches must be initialized depending upon the problem. Note that when the number of clauses of a problem increases, the connections become more complex.

## Results

**Figure 5:**
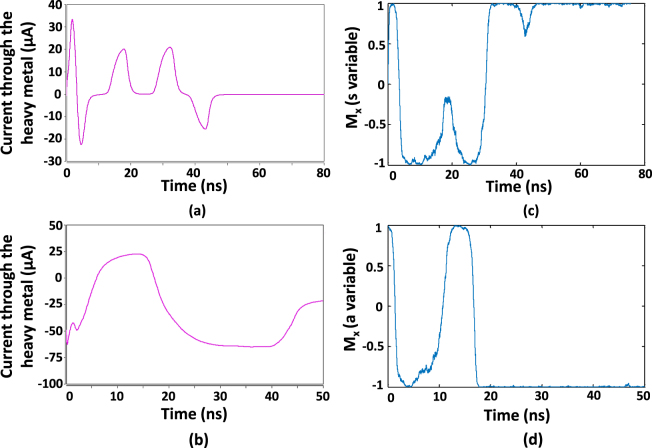
The varying current through the heavy metal layer of two MTJs that represent (**a**) *s* variable and (**b**) *a* variable of a 10-variable SAT instance (*α*_*c*_ = 4.25). The currents were obtained via HSPICE following the circuits explained in Fig. 4, simulated in IBM 45 nm technology. The resultant time evolution of the free layer magnetization along the $\hat{x}$ direction is shown on right for (**c**) *s* variable and (**d**) *a* variable.

In order to observe the functionality of our SAT solver, we conducted circuit level simulations. Figure 5 illustrates the currents through the heavy metal layers of the two MTJs representing *s* variable and *a* variable, that correspond to a 10-variable hard SAT instance. The results were obtained from HSPICE simulations using IBM 45 nm technology node. The resultant evolution of the free layer magnetization along the $\hat{x}$ direction (*M*_*x*_) is shown on the right (Fig. 5(c) and (d)). Note that a positive current drives the MTJ towards AP (+1) state and a negative current drives the MTJ towards P (−1) state in the figure.

**Figure 6:**
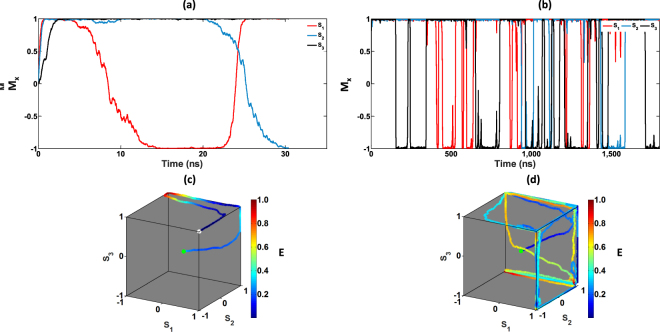
The time evolution of three variables in a 20 variable 3-SAT problem with different constraint densities. (**a**) and (**c**) correspond to a SAT problem with a constraint density *α*_*c*_ = 3 whereas (**b**) and (**d**) correspond to a SAT problem with a constraint density *α*_*c*_ = 4.25. (**c**) and (**d**) show the trajectories of the same 3 variables in (**a**) and (**b**) respectively, while converging to a solution inside a hypercube *Q*_3_. The colour presents the energy of the system at a given state. The starting point is a green circle and the end point (solution) is the vertex with a white circle.

As elaborated in the Supplementary section S1, the constraint density (*α*_*c*_) is an indicator of the hardness to solve a particular SAT instance. In order to observe the functionality of our solver for SAT instances with different hardness levels, we solved randomly generated 3-SAT problems with different constraint densities, and different number of variables. Figure 6 shows the magnetization dynamics of three MTJs that correspond to three variables in two 20 variable 3-SAT problems, each having a constraint density of 4.25 and 3.00, respectively. The colour of the trajectories in Fig. 6(c,d) indicates the normalized energy of the system at that particular point. This energy of the system can be defined by the following equations.

9 $$E(a,s)=\sum _{m=1}^{M}{a}_{m}{K}_{m}^{2}$$

10 $${K}_{m}={2}^{-k}\prod _{i=1}^{N}(1-{c}_{mi}{s}_{i})$$

where *M* and *N* are the number of clauses and the number of variables in the *k*-SAT problem, respectively. The energy is a function of the number of clauses not satisfied at a particular instant. This can be used as a cost function to determine the “satisfiedness” of a particular problem at a given instant. Notice that the trajectories in Fig. 6(c),(d) pass through higher energy states as the system tries to converge to a solution. This shows that our system escapes local minimum points naturally, unlike other algorithms^28^ where simulated annealing is necessary to escape from such local minimum points.

### Approximate polynomial time solution from the proposed SAT solver

**Figure 7:**
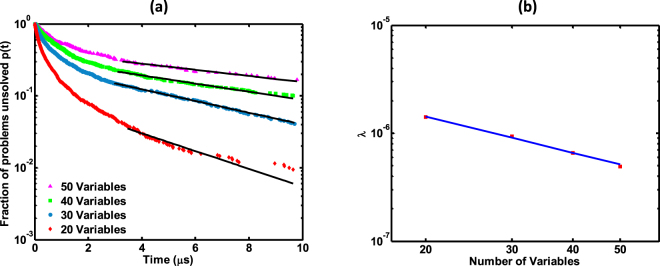
Computation time of the SAT solver (**a**) The fraction of problems *p*(*t*) not yet solved at real time *t*, for 3-SAT problems with *α*_*c*_ = 4.25, for *N* = 20, 30, 40 and 50. Averages were calculated with 10^3^ instances for each *N*. (**b**) The decay rate *λ* for different number of variables. *λ* takes the form *λ*(*N*) = *bN*^−*β*^ with *β* approximately 1.1 (note the log scale).

We also solved randomly generated satisfiable 3-SAT problems in the hard regime (*α*_*c*_ = 4.25) for different number of variables (20, 30, 40, 50). We have calculated the number of problems solvable within 10 *μ*s for the purpose of illustration. However, since our system has no limit cycles owing to thermal noise (more details are available in the next section), we argue that our proposed method will probably reach a solution if sufficient time has been provided, given that a solution exist. We monitored the fraction of problems not solved by the algorithm at time *t* and the result is depicted in Fig. 7. It is evident that the fraction of problems not solved *p*(*t*), has an exponential decay with time *t*. The relationship between *p*(*t*) and *t* can be approximated by

11 $$p(t)=r{e}^{-\lambda (N)t+\gamma }$$

where *r* and *γ* are constants. The decay rate *λ* obeys *λ*(*N*) = *bN*^−*β*^, with *β* ≈ 1.1. Therefore the continuous time *t* needed to solve a (1−*p*) fraction of problems can be written as

12 $$t(p,N)=(\gamma +\,{\rm{l}}{\rm{n}}(r/p)){b}^{-1}{N}^{\beta }$$

This implies that the time to solve a (1−*p*) fraction from a set of *k*-SAT problems is of polynomial complexity (for the range of N we have considered). This polynomial relationship still holds when the fraction of problems left unsolved is a fixed number (irrespective of the number of variables)^8^. That is, the time taken to solve all possible *k*-SAT formulae for a given *N* and *α*_*c*_ (Θ(*k*, *N*, *α*_*c*_)), except for a constant amount of problems *c* (*p*(*t*) = *c*/Θ(*k*, *N*, *α*_*c*_)), would follow a relationship as shown below when *N*→∞^8^ (if we assume that the relationship in equation (11) and (12) still holds as *N*→∞).

13 $$t(p,N)\sim {N}^{\beta +1}ln(N)$$

A previous proposal^8^ shows a similar relationship for the time required to solve *k*-SAT problems. However, the relationship is not valid in real time if a digital computer is to be used for the calculations. It is because, the complexity of the analog system varies over time, and when solving each step, it would take different real time values depending upon the complexity. Since our method is purely based on hardware, we argue that the above approximate polynomial time relationship is valid in real time for our proposed system. We conjecture that this behaviour is due to multiple reasons including the thermal noise associated with the MTJs. It has also been predicted^9^ that, the noise effects may avoid long transient oscillations. Our results too suggest that higher amounts of noise leads to faster convergence (explained in the next section). It must be noted that, this proposed MTJ based SAT solver does not behave identical to the cellular neural network based solver in equations (2–3). Our solver has some added complexities not present in the CNN based system (refer to the set of equations in Supplementary section S4). We conjecture that such complexities offered by the device physics, acts favorable to give faster solutions to SAT problems as well. However, these benefits come at the cost of handling the circuit limitations (fan-out etc.) that can arise when solving problems with larger *N*.

### Effect of thermal noise

Thermal noise has significant impact on the switching dynamics of nano-magnets. Equation 14 explains the renowned Brown’s model^29^ that captures the behaviour of thermal noise which can be used as a random magnetic field in the LLGS equations.

14 $${\overrightarrow{H}}_{Thermal}=\overrightarrow{\varsigma }\sqrt{\frac{2\alpha {k}_{B}T}{|\gamma |{M}_{s}V}}$$

$\overrightarrow{\varsigma }$ is a vector with components that are zero mean, unity standard deviation, Gaussian random variables. *V* is the volume of the free layer, *T* is the temperature, and *k*_*B*_ is the Boltzmann’s constant. The time discretization value *dt* must be included in the numerator when solving the equation numerically. The existence of thermal noise is mandatory for the proper operation of our SAT solver. This is due to the tilt of the FL magnetization with the easy axis, induced by thermal noise without which a magnet cannot be switched. Results indeed show that, under zero thermal noise, the magnetizations evolve to frozen non-solution states (Supplementary section S5). In the next two subsections, we will explain how the thermal noise assists in avoiding limit cycles and the impacts of larger thermal noise on the time to converge to a solution.

#### Not behaving as a chaotic dynamic system and absence of limit cycles

We define the states of all the MTJs that represent variable *s* as *H*_*N*_ = [−1, 1]^*N*^. The P state is mapped to −1 and the AP state is mapped to +1. The boundary of *H*_*N*_ is the *N* hypercube *Q*_*N*_, with vertices *V*_*N*_ = {−1, 1}^*N*^. The solution space to a particular problem can be a subset of these *V*_*N*_. Let us denote such a solution by ${{V}_{N}}^{sol}=\{{{V}_{N}}^{i},{{V}_{N}}^{j},\mathrm{...}\}$. Due to the effect of thermal noise, the solution to which the system will ultimately converge has minimal dependency with the initial states of the MTJs. For example, let us consider a SAT problem that has multiple solutions ${V}_{N}^{sol}$ and solving it in two trials with the same initial states of MTJs. The output solutions in the two trials may not be the same even though the starting conditions were identical. This implies that our system does not show any chaotic behaviour (it is not deterministic) in contrast to the system given by equations 2 and 3.

If the states of the MTJs that represent variable *s* continuously change in a periodic manner (it has entered a limit cycle), it is possible that the system never reaches a solution (even if there exists one). That is, *s*_*i*_(*t*) = *s*_*i*_(*t* + *nT*) for ∀*n* = 1, 2, … and *s*_*i*_(*t*) is not a solution of the system. This is known as the system getting trapped in a limit cycle^8^. It is shown that for the system explained in equations 2–3, this can occur for certain coupling parameter (*A*, *B*) choices^9^. However, due to the added stochasticity from the thermal noise, we argue that our MTJ based SAT solver does not get trapped in limit cycles. It is highly probable that our system reaches a solution if sufficient amount of time is provided.

#### Increasing temperature resulting in faster solution convergence

**Figure 8:**
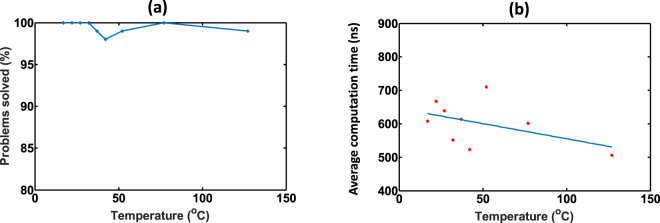
(**a**) The percentage of randomly generated 20 variable SAT problems (*α*_*c*_ = 4.25) solved at different temperatures within 10 *μs*. (**b**) Average time to solve a 20 variable SAT problem at different temperatures.

Now let us consider how different amounts of thermal noise will affect the operation of our MTJ based SAT solver. Temperature can affect the amount of thermal noise applied on an MTJ device (equation 14). Sufficiently higher temperatures on MTJs with smaller switching energy barriers, can cause the state of an MTJ to oscillate over time, even without any input current or magnetic field^25^. We solved randomly generated 20 variable, 3-SAT problems at different temperatures and observed the percentage of problems that can be solved within 10 *μs*. As Fig. 8(a) illustrates, it is evident that in the range of 20°*C*−130°*C*, there is no significant degradation in the percentage of problems solved within 10 *μs*. However, according to Fig. 8(b), it appears that the average time to solve a *k*-SAT problem decreases by ∼100 *ns* with increasing temperature in the range 20°*C*–130°*C*.

### Effects of process variations

In our design, we selected a particular thickness (*t*_*ss*_) for the free layers of the MTJs. In reality, it is impossible to achieve the exact thickness due to number of reasons including atomic limitations, process variations, fabrication limitations *etc*. How much precession is present along the easy axis is dependent upon how much the actual thickness has deviated from *t*_*ss*_. In order to observe the effects of thickness variations on the operation of our proposed SAT solver, we consider two scenarios.

1. Global variation of thickness.
2. Local variation of thickness.

**Figure 9:**
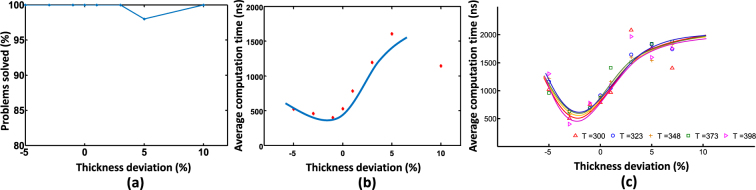
(**a**) The percentage of randomly generated 20 variable SAT problems (*α*_*c*_ = 4.25) solved at different percentage variations in thickness (from the seamless switching thickness, *t*_*ss*_) of the free layer within 10 *μs*. (**b**) Average time to solve a 20 variable SAT problem at different percentage variations in thickness of the free layer. The variations are considered as global. *i*.*e*., all the devices in the SAT solver has the thickness with same deviation from *t*_*ss*_ (**c**) Same experiment in (**b**) performed for different temperatures. Temperature indicated in Kelvin.

In the first case, we perturb all the thicknesses of MTJs in our system by some constant percentage from *t*_*ss*_. For a particular percentage global variation in thickness, we solved randomly generated 20 variable, SAT problems. Then the percentage of problems solvable within 10 *μs*, and the average time to solve a single problem was observed. Figure 9(a) illustrates that there is no significant change in the percentage of solvable problems when the global variations in thickness is changed from −5% to +10%. However, as Fig. 9(b) shows, the average convergence time increases when the deviations in thickness increases. We also observed that the solver no longer works if the thickness is less than a particular value. This is the limit at which the perpendicular magnetic anisotropy (PMA) becomes dominant and the FL magnetization stabilizes in $\hat{z}$ axis instead of $\hat{x}$ axis. We observed this when the actual thickness is ∼−10% deviated from the *t*_*ss*_, for the choice of materials and dimensions used in this work. In the second case, we change thicknesses of all the MTJs according to a Gaussian distribution with a 3*σ* value (where *σ* is the standard deviation) of 10% from the *t*_*ss*_. The solver gave an average convergence time of 588.31*ns* and was able to solve 97% of randomly generated 20 variable, 3-SAT instances within 10 *μs*.

As described in the previous section, the temperature increments inversely affect the computation time of the MTJ based system. To see the effect of both thickness and temperature variations simultaneously, we conducted the above experiment at different temperatures. The results are summarized in Fig. 9(c) and it shows that the changes in computation time due to thickness deviation is more prominent than that due to temperature drift.

During the fabrication process, other non-idealities such as edge damages^30,31^ can be present in the MTJs. We introduced variations in interface anisotropy constant, width and length of the free layer of MTJs, to observe the effect of aforementioned non-idealities. The effects of anisotropy constant variations on computation time follow a similar trend as the ‘computation time vs thickness’ curve (Fig. 9). We further noticed that the computation time decreases when the free layer dimensions decrease. The percentage of problems solved remained almost at 100%. More detailed results are included in the Supplementary section S6.

### Power consumption and computation time of the SAT solver

In this section, we will present the power consumption and computation time of our proposed system, and compare with existing methodologies. In order to calculate the power consumption, we used SPICE simulations in IBM 45 nm technology. The measured average power consumption over solving 1,000, 20-variable (our software based LLGS solving framework could not handle bigger benchmarks such as ‘ais8’^32^) hard SAT problems (constraint density *α*_*c*_ = 4.25) was 1.37 mW. The power requirements of each section of the solver are presented in Table 1. We observed that the peripheral circuits such as amplifiers and voltage controlled current drivers consume a significant portion of power. The power consumption of the total MTJ and heavy metal layer structures is approximately about 20% of the total power consumption of the system. Similarly, a recent work of a hardware realization^16^ of an analog approach^8^, also explains that their overall power consumption to be significant (numerical value not specified), due to the usage of op-amps. In another design^15^, the CNN based SAT solving algorithm^9^ used in our work was realized using op-amp based integrators. The power consumption was reported as 140 *μ*W for a 4-variable, 4-clause problem. For comparison, we evaluated the average power consumption of our MTJ based solver for similar 4-variable 4-clause SAT instances, and witnessed a power consumption of 84 *μ*W, which is about 40% smaller than the aforementioned analog hardware design^15^.

**Table 1:** Power consumption of the MTJ based SAT solver.

Component	Power consumption
Read circuitry	300 nW
Amplifiers	315 *μ*W
Voltage controlled HM current drivers	827 *μ*W
Driving current through HM	235 *μ*W
Total	1377 *μ*W

Digital hardware for realizing typical SAT solving algorithms such as GRASP^11^, DPLL^33^ *etc*. can be found in literature^34–36^. A custom IC^34^ designed for such an algorithm reported a power consumption of 871 *μ*W. This is about 37% lower than our design. However, these digital approaches are slower than analog solvers due to the step-by-step decision making and backtracking required for solving a problem. It is also noteworthy that our proposal is an asynchronous method in contrast to the above synchronous methods. Therefore, the need for a clock signal and the power associated with it is not present in our design. Our system automatically goes to a minimum energy point (which is a solution) and stabilizes there as explained mathematically in Supplementary section S4.

In order to obtain the computation time of our system, we conducted system level simulations using random 1,000, 20-variable hard SAT instances. The time taken to solve all 1,000 problems (100%) were evaluated and the average computation time per instance was 553 ns. This computation time was then compared with a state-of-the-art software SAT solver, Minisat^37^. The same 1,000 problems were solved using Minisat in a 3.6 GHz processor and the average computation time was evaluated (1.44 ms). We observed an average speed-up of 2.6×10^3^ with respect to Minisat. Prior hardware SAT solver designs in literature have also presented their speed-up with respect to Minisat, and the values are summarized in Table 2 for comparison. Note that the Boolean Constraint Propagation (BCP) accelerator^38^ in Table 2 has reported the speed up with respect to a purely software based algorithm (modified zChaff^10^) which has the same performance for BCP when compared with Minisat. Furthermore, the analog CNN based system^9^ built using op-amp based integrators^15^ has an average computation time of 15 *μ*s for 10 variable, 2-SAT problems. Our MTJ based proposal has an average run time (over 1,000 instances) of 186 ns for 10 variable 3-sat (*α*_*c*_ = 4.25) problems (showing that the MTJ based solver is ∼30× faster).

**Table 2:** Speed-up of hardware based SAT solvers with respect to purely software based solvers.

Hardware solver	Speed-up with respect to software based solvers
Reconfigurable SAT solver^35^	90 (3.6 GHz processor)
BCP accelerator^38^	6.7 (3.6 GHz processor)
BCP accelerator^36^	4 (3.3 GHz processor)
MTJ based solver	2.6 × 10^3^ (3.6 GHz processor)

## Conclusion

Boolean satisfiability is an NP-complete problem (k ≥ 3) that finds utility in vast array of applications^1–5^. Analog solutions to the satisfiability problem has recently appeared attractive^8,9,15^ due to the massive parallelism available when solving, in contrast to the stepwise search algorithms. In this work, we provide a proof of concept hardware based analog SAT-solver using Magnetic Tunnel Junctions driven by the Spin Orbit Torque Phenomenon. We have mathematically shown how the inherent device physics of MTJs closely mimics an existing analog approach^9^ to solving the Boolean satisfiability problem. Device and circuit level simulations were conducted to solve hard satisfiability problems in order to observe the performance and functionality of our proposed system. According to the observations, we witnessed that the proposed SAT solver is capable of finding a solution to a significant fraction (>85%) of hard SAT problems in polynomial time. We conjecture that this is due to the inherent thermal noise present in MTJs and the device complexities added on top of the existing analog approach^9^. The SAT solver automatically comes out of local minimum points and limit cycles due to thermal noise. Therefore, it is highly probable that the system reaches a solution if sufficient time is provided, given that the SAT problem has a solution. Further, the variation analysis illustrates that our proposed solver is robust to variations in the MTJ thickness in the range of −5% to 10%. Larger variations result in higher average convergence time. The proposed MTJ based SAT solver is 2.6 × 10^3^ times faster than a state-of-the-art software solver, Minisat.

## Methods

The set of equations involved in modelling the HM-MTJ structures is provided in Supplementary sections S2, S3 and S4. The *t*_*ss*_ of the MTJs was calculated by self consistently solving the equation (8) and the analytical equations for the demagnetization factors^39^. IBM 45 nm technology node was used to simulate the CMOS interface circuitry. The material parameters (selected according to the experimental papers^24,40^) and the device dimensions are summarized in supplementary Table S1.

## Electronic supplementary material

Supplementary file

## References

[1] Bao, F. S. *et al*. Accelerating boolean satisfiability (sat) solving by common subclause elimination. *Artificial Intelligence Review* 1–15 (2017).

[2] Saha, S. *et al*. Improved test pattern generation for hardware trojan detection using genetic algorithm and boolean satisfiability. In *International Workshop on Cryptographic Hardware and Embedded Systems*, 577–596 (Springer, 2015).

[3] Vizel Y, Weissenbacher G, Malik S (2015). Boolean satisfiability solvers and their applications in model checking. Proceedings of the IEEE.

[4] Stanley J, Liao H, Lafortune S (2015). Sat-based control of concurrent software for deadlock avoidance. IEEE Transactions on Automatic Control.

[5] Chung, Y.-T. & Jiang, J.-H. *Functional timing analysis method for circuit timing verification US Patent 8*, **671**, 375 (2014).

[6] Karp, R. M. Reducibility among combinatorial problems. In *Complexity of computer computations*, 85–103 (Springer, 1972).

[7] Cook, S. A. The complexity of theorem-proving procedures. In *Proceedings of the third annual ACM symposium on Theory of computing*, 151–158 (ACM, 1971).

[8] Ercsey-Ravasz M, Toroczkai Z (2011). Optimization hardness as transient chaos in an analog approach to constraint satisfaction. Nature Physics.

[9] Molnár B, Ercsey-Ravasz M (2013). Asymmetric continuous-time neural networks without local traps for solving constraint satisfaction problems. PloS one.

[10] Moskewicz, M. W. *et al*. Chaff: Engineering an efficient sat solver. In *Proceedings of the 38th annual Design Automation Conference*, 530–535 (ACM, 2001).

[11] Marques-Silva JP, Sakallah KA (1999). Grasp: A search algorithm for propositional satisfiability. IEEE Transactions on Computers.

[12] Paturi R, Pudlák P, Saks ME, Zane F (2005). An improved exponential-time algorithm for k-sat. Journal of the ACM (JACM).

[13] Guo, L., Hamadi, Y., Jabbour, S. & Sais, L. Diversification and intensification in parallel sat solving. *Principles and Practice of Constraint Programming–CP* 2010 252–265 (2010).

[14] Siegelmann, H. T. Computation beyond the turing limit. In *Neural Networks and Analog Computation*, 153–164 (Springer, 1999).

[15] Basford, D. A. *et al*. The impact of analog computational error on an analog boolean satisfiability solver. In *Circuits and Systems (ISCAS), 2016 IEEE International Symposium on*, 2503–2506 (IEEE, 2016).

[16] Yin X (2018). Efficient analog circuits for boolean satisfiability. IEEE Transactions on Very Large Scale Integration (VLSI) Systems.

[17] Fan D, Sharad M, Sengupta A, Roy K (2016). Hierarchical temporal memory based on spin-neurons and resistive memory for energy-efficient brain-inspired computing. IEEE transactions on neural networks and learning systems.

[18] Sengupta A (2016). Magnetic tunnel junction mimics stochastic cortical spiking neurons. Scientific reports.

[19] Sengupta A, Shim Y, Roy K (2016). Proposal for an all-spin artificial neural network: Emulating neural and synaptic functionalities through domain wall motion in ferromagnets. IEEE transactions on biomedical circuits and systems.

[20] Behin-Aein B, Datta D, Salahuddin S, Datta S (2010). Proposal for an all-spin logic device with built-in memory. Nature nanotechnology.

[21] Wijesinghe, P., Ankit, A., Sengupta, A. & Roy, K. An all-memristor deep spiking neural computing system: A step towards realizing the low power, stochastic brain. *arXiv preprint arXiv:1712.01472v3* (2017).

[22] Yu G (2014). Switching of perpendicular magnetization by spin-orbit torques in the absence of external magnetic fields. Nature nanotechnology.

[23] Liu L (2012). Spin-torque switching with the giant spin hall effect of tantalum. Science.

[24] Pai C-F (2012). Spin transfer torque devices utilizing the giant spin hall effect of tungsten. Applied Physics Letters.

[25] Liyanagedera CM, Sengupta A, Jaiswal A, Roy K (2017). Stochastic spiking neural networks enabled by magnetic tunnel junctions: From nontelegraphic to telegraphic switching regimes. Physical Review Applied.

[26] Fong, X. *et al*. Knack: A hybrid spin-charge mixed-mode simulator for evaluating different genres of spin-transfer torque mram bit-cells. In *Simulation of Semiconductor Processes and Devices (SISPAD), 2011 International Conference on*, 51–54 (IEEE, 2011).

[27] Slonczewski JC (1989). Conductance and exchange coupling of two ferromagnets separated by a tunneling barrier. Physical Review B.

[28] Jonke, Z., Habenschuss, S. & Maass, W. Solving constraint satisfaction problems with networks of spiking neurons. *Frontiers in neuroscience* **10** (2016). 10.3389/fnins.2016.00118 PMC4811945 27065785

[29] Brown WF (1963). Thermal fluctuations of a single-domain particle. Physical Review.

[30] Song K, Lee K-J (2015). Spin-transfer-torque efficiency enhanced by edge-damage of perpendicular magnetic random access memories. Journal of Applied Physics.

[31] Sun Z, Retterer S, Li D (2014). The influence of ion-milling damage to magnetic properties of co80pt20 patterned perpendicular media. Journal of Physics D: Applied Physics.

[32] Hoos HH, Stützle T (2000). Satlib: An online resource for research on sat. Sat.

[33] Davis M, Logemann G, Loveland D (1962). A machine program for theorem-proving. Communications of the ACM.

[34] Gulati, K. & Khatri, S. P. Accelerating boolean satisfiability on a custom ic. In *Hardware Acceleration of EDA Algorithms*, 33–61 (Springer, 2010).

[35] Gulati K, Paul S, Khatri SP, Patil S, Jas A (2009). Fpga-based hardware acceleration for boolean satisfiability. ACM Transactions on Design Automation of Electronic Systems (TODAES).

[36] Thong, J. & Nicolici, N. Fpga acceleration of enhanced boolean constraint propagation for sat solvers. In *Proceedings of the International Conference on Computer-Aided Design*, 234–241 (IEEE Press, 2013).

[37] Eén, N. & Sörensson, N. Minisat 2.2. http://minisat.se (2013).

[38] Davis, J. D., Tan, Z., Yu, F. & Zhang, L. A practical reconfigurable hardware accelerator for boolean satisfiability solvers. In *Design Automation Conference, 2008. DAC 2008. 45th ACM/IEEE*, 780–785 (IEEE, 2008).

[39] Aharoni A (1998). Demagnetizing factors for rectangular ferromagnetic prisms. Journal of applied physics.

[40] Ikeda S (2010). A perpendicular-anisotropy cofeb–mgo magnetic tunnel junction. Nature materials.

[41] Sakuraba Y (2010). Co-concentration dependence of half-metallic properties in co–mn–si epitaxial films. Applied Physics Letters.

[42] Yuasa S, Djayaprawira D (2007). Giant tunnel magnetoresistance in magnetic tunnel junctions with a crystalline mgo (0 0 1) barrier. Journal of Physics D: Applied Physics.

