# Supplementary material for: Analog Approach to Constraint Satisfaction Enabled by Spin Orbit Torque Magnetic Tunnel Junctions
Source: Sci Rep. 2018 May 2;8:6940. doi: 10.1038/s41598-018-24877-z (PMC5932068; doi:10.1038/s41598-018-24877-z)
Supplement: Supplementary file 1 — Supplementary file [file 41598_2018_24877_MOESM1_ESM.pdf]

Analog Approach To Constraint Satisfaction Enabled By Spin Orbit Torque Magnetic Tunnel Junctions

Supplementary Information

Parami Wijesinghe, Chamika Liyanagedera & Kaushik Roy

School of Electrical and Computer Engineering,
Purdue University, West Lafayette, Indiana 47907, USA

S1: Cellular Neural Network (CNN) based continuous time dynamical system for a SAT solver

The analog Cellular Neural Network (CNN) based SAT solver can be defined by the following set of equations[1] (same as the main article).

$$\dot{s}_i(t) = \frac{ds_i(t)}{dt} = -s_i(t) + Af(s_i(t)) + \sum_m c_{mi} g(a_m(t)) \tag{S1}$$

$$\dot{a}_m(t) = \frac{da_m(t)}{dt} = -\,a_m(t) + Bg(a_m(t)) - \sum_i c_{mi} f(s_i(t)) + 1 - k \tag{S2}$$

The functions $f()$ and $g()$ are the thresholding functions applied on variables s_i and a_m, respectively, as shown in figure S1.

The variable s_i represents the state of the ith $(i = 1, 2, ..., N)$ Boolean variable (x_i) and a_m represents the "satisfiedness" of mth $(m = 1, 2, ..., M)$ clause of the Boolean function. When the Boolean variable is true $(x_i = 1)$, then the value of s_i after thresholding will be equal to 1 (*i.e.* $f(s_i) = 1$). When it is false $(x_i = 0)$, then $f(s_i) = -1$. The vector $f(s)$ can be considered as a solution to the k-SAT problem when all the transient variations have stopped and the system is in a stable state. The variable a_m determines whether the mth $(m = 1, 2, ...M)$ clause is satisfied at a given moment depending upon the values of the s variables. When the value of variable a_m after thresholding is 0 (*i.e.* $g(a_m) = 0$), then the corresponding m^{th} clause is satisfied. It will not have

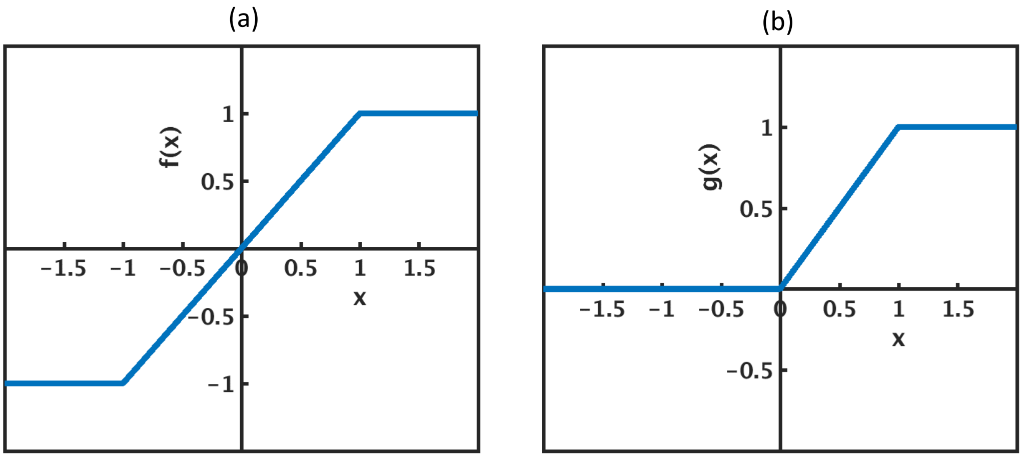

Figure S1: The thresholding functions (a) $f()$ and (b) $g()$ of the analog SAT solver defined by equations (S1) and (S2)

any impact on the dynamics of the system afterwards. The system converges to a solution of the Boolean function (get stabilized), when vector $g(a)$ is zero. The parameters A and B ('self-coupling parameters') are constants and C is a matrix of size (M, N) that is unique to a given propositional formula $\mathcal{F}$ (main article, equation 1). The elements of the c matrix can be defined as follows. C_m is the mth clause.

$$c_{mi} = \begin{cases} 1 & if\ x_i \in C_m \\ -1 & if\ \overline{x}_i \in C_m \\ 0 & if\ x_i \notin C_m\ and\ \overline{x}_i \notin C_m \end{cases} \tag{S3}$$

The constraint density (α_c) can be considered as a measure of hardness of a k-SAT problem and is defined as the ratio between the number of clauses and the number of variables

$$\alpha_c = M/N \tag{S4}$$

In the easy SAT region, only few constraints are there to be satisfied leading to a small α_c. When the number of clauses in the problem is large, deciding whether the propositional formula is unsatisfiable (UNSAT) is easy. There is an intermediate range (hard SAT), where solving the satisfiability problem can be challenging. It has been shown that, for 3-SAT, the hardest problem regime is when the constraint density α_c of the Boolean function is 4.25 and for a 4-SAT problem, the hardest regime is at α_c=9.55 *etc.*[1–4] Also, the worst-case complexity of any k-SAT problem depends exponentially on the number of variables, N.[5] The above explained CNN based system gives solutions to k-SAT problems even in their hardest regime. However, proper tuning of self-coupling parameters A and B is indispensable to achieve better performance. Figure S2 depicts the time evolution of s and a variables when solving a 3-sat problem with 10 variables, using proper A, B values for equations (S1-S2).

S2: Free layer magnetization dynamics of an MTJ

A Magnetic Tunnel Junction (MTJ) stack is composed of two ferromagnetic layers called the pinned layer (PL) and the free layer (FL), separated by a thin tunneling oxide layer (MgO). The pinned layer is magnetically hardened to have a fixed magnetization direction ($\widehat{p}$) and acts as a reference layer. On the other hand, the magnetization direction $\widehat{m}$ of the free layer can be switched by passing a charge current through either the MTJ itself or through a heavy metal (HM) underlayer. It is also possible to switch an MTJ using an external magnetic field. The magnetization motion when switching due to a current or an external field can be explained using the following Landau-Lifshitz-Gilbert-Slonczewski (LLGS) equation.[6]

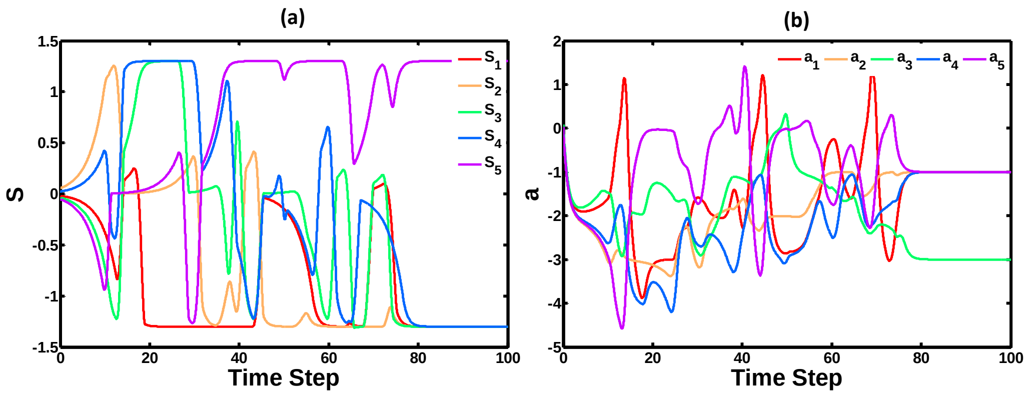

Figure S2: The time evolution of s and a (in (a) and (b) respectively) variables when solving a random 3-SAT problem with 10 variables using the system defined by equations (S1) and (S2). The constraint density (α_c) of the problem is 4.25. The self-coupling parameters A and B are set to 1.3 and 2.3 respectively. Only dynamics of five variables are shown here for clarity. The system has converged to a solution at the 80th time step. Note that $g(a)$ is zero after this time.

Table S1: Device simulation parameters

Parameters	Values
Free layer area, A_{MTJ}	20×40 nm^2
Free layer thickness, t_{ss}	1.72nm
Gilbert's damping factor, α	0.02
Saturation Magnetization, M_S	1257.3 KA/m
Interface Anisotropy, K_i	1.3 ergs/cm^2
Spin hall angle, θ_{SH}	0.3
Heavy metal layer thickness	3 nm
Heavy metal layer resistance	50Ω
Oxide thickness, t_{ox}	1.5nm
Tunnel Magneto-Resistance, (TMR)	180%
Switching current, (full switch, $AP \to P$, 20ns)	17μA

$$\frac{(1+\alpha^2)}{|\gamma|}\frac{\partial \widehat{m}}{\partial t} = -\widehat{m} \times \overrightarrow{H}_{EFF} - \alpha \widehat{m} \times \widehat{m} \times \overrightarrow{H}_{EFF} + \frac{1}{|\gamma|}(\alpha \widehat{m} \times \overrightarrow{STT} + \overrightarrow{STT}) \tag{S5}$$

$$\widehat{m} = [m_x\widehat{x} \quad m_y\widehat{y} \quad m_z\widehat{z}] \tag{S6}$$

where α is the Gilbert damping constant, γ is the gyro-magnetic ratio, $\widehat{m}$ is the unit vector in the direction of the magnetization, t is the continuous time, and $\overrightarrow{H}_{EFF}$ is the effective magnetic field including the demagnetization field and the interface anisotropy field. ($\overrightarrow{STT}$) is the spin transfer torque term given by

$$\overrightarrow{STT} = |\gamma|\beta\Big(\widehat{m} \times (\epsilon\widehat{m}) \times \widehat{p}\Big), \quad \beta = \frac{\hbar J_s}{2q\mu_0 M_s t_{FL}} \tag{S7}$$

where q is the charge of an electron, μ_0 is vacuum permeability, $\hbar$ is the modified Planck's constant, t_{FL} is the thickness of FL and M_s is the saturation magnetization. J_s is the spin current density incident on the free layer of the MTJ and ϵ is the spin polarization efficiency. The relationship between this spin current and the charge current will be explained in section S4. For this work, all the material parameters were selected according to prior experimental work.[7,8] These parameters along with the device dimensions are recorded in Table S1.

S3: The derivation of thickness for dominant in plane magnetic anisotropy MTJ devices.

The state of an MTJ at a particular time instant depends on its previous state, and the current that passes through the the HM layer. In order to mimic the CNN based dynamic system (equation S1-S2), it is important that the state of an MTJ (when there is no applied current) goes to the parallel (anti-parallel) state, if the angle between the FL and the PL magnetization is $\theta_{fp} < \pi/2$ ($\theta_{fp} > \pi/2$). We have optimized the thickness of the FL so that the MTJ exhibits this behavior. Figure S3 shows how a typical MTJ with a dominant in-plane magnetic anisotropy field (IMA-MTJ) behaves (here the actual thickness is larger than the value we have derived). The color of each point in the graph refers to the ultimate state to which the free layer magnetization settles down with no current applied. The position of each point shows the initial condition [ϕ,θ] in standard spherical coordinate notations.

We will now explain how we can make the MTJ state go towards the parallel (anti-parallel) state when $\theta_{fp} < \pi/2$ ($\theta_{fp} > \pi/2$) at a particular time instant after which there is no driving current. Let us first consider the fields that affect the FL magnetization. The $\overrightarrow{H}_{EFF}$ in the LLGS equation (S5) consists of two major types of anisotropy fields; the demagnetization field and interface anisotropy field. For typical choices of materials for MTJs, these anisotropy fields are of the following form.

Demagnetization field[9]

$$H_D = [-M_sN_{xx}m_x \quad -M_sN_{yy}m_y \quad -M_sN_{zz}m_z] \tag{S8}$$

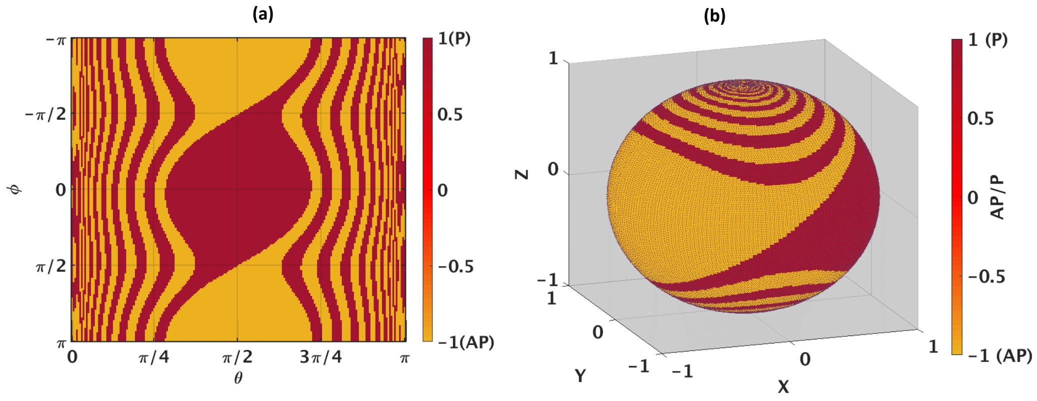

Figure S3: The two colours show the state to which the free layer magnetization settles down in a typical IMA-MTJ (FL thickness $\neq t_{ss}$ in equation (S11)). There is no current or external field applied, and the initial condition is (ϕ, θ) in standard spherical coordinate notations. Note that thermal noise is not present in this analysis. The pinned layer magnetization is in $+\hat{x}$ direction. It is evident that the angle between the free and pinned layer (θ_{fp}) does not alone decide the final state of the MTJ. (b) shows the same final states in (a) in 3-dimensional space.

Interface anisotropy field[10]

$$H_\perp = \begin{bmatrix} 0 & 0 & \frac{2K_i}{\mu_0 M_s t_{FL}} m_z \end{bmatrix} \tag{S9}$$

Where N_{xx}, N_{yy} and N_{zz} are the demagnetization factors, and K_i is the energy density constant for the perpendicular interface anisotropy. When the applied current to the MTJ through the HM layer is zero, the current induced spin transfer torque will not be present (i.e. $\overrightarrow{STT} = 0$). The component of the magnetization that contributes to the resistance is the one along the direction of the fixed layer (or easy axis which is $\hat{x}$ in this particular case). The dynamics of that component of magnetization is as follows.

$$\begin{aligned}
\left(\frac{1+\alpha^2}{|\gamma|}\right)\frac{\partial m_x}{\partial t} &= -\left(\hat{m} \times \vec{H}_{EFF} + \alpha \hat{m} \times \hat{m} \times \vec{H}_{EFF}\right).\hat{x} \\
&= -\left(m_y H^z_{EFF} - m_z H^y_{EFF} - \alpha|\hat{m}|^2 H^x_{EFF} + \alpha m_x \left(m_x H^x_{EFF} + m_y H^y_{EFF} + m_z H^z_{EFF}\right)\right) \\
&= -\Bigg(- m_y m_z N_{zz} M_s + m_y m_z N_{yy} M_s + \frac{2K_i}{\mu_0 M_s t_{FL}} m_y m_z \\
&\quad + \alpha\Big((1-m_x^2) N_{xx} M_s m_x - m_x m_y^2 N_{yy} M_s - m_x m_z^2 N_{zz} M_s \quad + \frac{2K_i}{\mu_0 M_s t_{FL}} m_x m_z^2\Big)\Bigg)
\end{aligned} \tag{S10}$$

According to the above equations, it is evident that the final state of the free layer magnetization does not solely depend on the sign of m_x. The existence of terms such as $m_y m_z$ suggests that the final state will depend even on m_y and m_z as well, justifying the behavior depicted by figure S3. To address this, we use a FL thickness as follows (similar to equation 8 in the main article).

$$t_{ss} = \frac{2K_i}{(N_{zz} - N_{yy})\mu_0 M_s^2} \tag{S11}$$

This thickness t_{ss} will make the $\hat{x}$ component of the precession term $(\hat{m} \times \vec{H}_{EFF}).\hat{x}$ zero in the LLGS equation. The updated magnetization dynamics is shown in equation S12. Note that during the derivation we used the fact that $N_{zz} > N_{yy} > N_{xx}$. This is due to the special dimension requirement that must be followed to allow the magnetization to be stable in the $\hat{x}$ direction. The aspect ratio between the length and the width must be greater than one, as graphically shown in figure 1 in the main article.

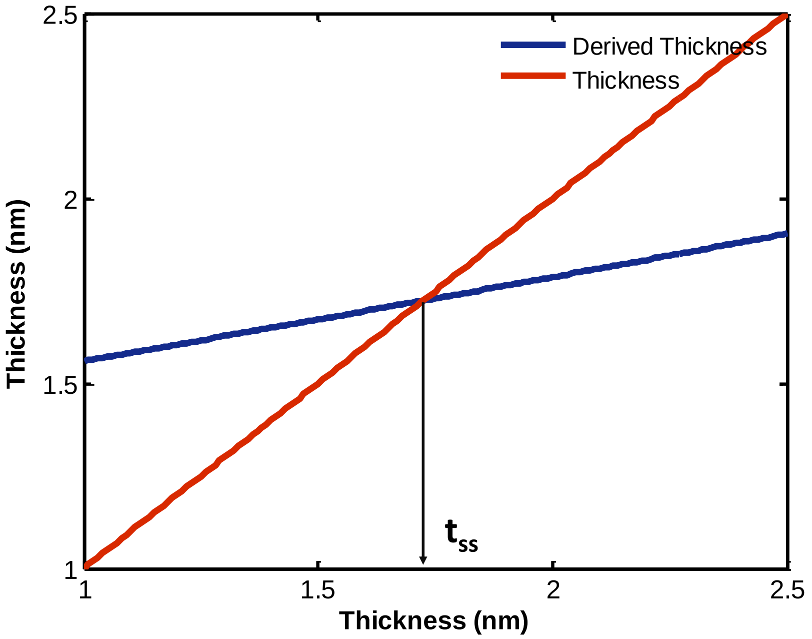

Figure S4: Seamless switching thickness (t_{ss}) solved self consistently for a rectangular IMA-MTJ device. "Derived thickness" (blue) is the t_{ss} obtained from equation (S11) with demagnetization parameters that correspond to the thickness in x axis. "Thickness" (red) is the 1:1 graph of FL thickness.

$$\left(\frac{1+\alpha^2}{|\gamma|}\right)\frac{\partial m_x}{\partial t} = m_x \alpha M_s (1-m_x^2)(N_{yy}-N_{xx}) \tag{S12}$$

Now the dynamics depict a direct relationship with the sign of m_x. When $m_x > 0$, ($m_x < 0$) the final state will be parallel (antiparallel) when no spin current is present, provided that the PL magnetization is directed towards $+\hat{x}$ direction. The effect of noise and process variations is not considered in this section. It is separately discussed in the main article. Note that the demagnetization factors N_{zz}, N_{yy} (in equation S11) in return depends on the dimensions of the FL including the thickness.[11] Therefore, solving for the t_{ss} must be done self consistently. Figure S4 shows the evaluation of this t_{ss} (or the 'seamless switching' thickness as explained in the main article) for a particular MTJ device.

The new FL final magnetization states of an MTJ with the optimized thickness are graphically shown in figure S5. It illustrates how the magnetization converges to a state under zero drive current when the free layer thickness is t_{ss}. Note that the final magnetization depends on just whether m_x is positive or negative, at the time the write current is made zero. The effect of noise will make the final state, a probabilistic function of the magnitude of m_x. *i.e.* higher the magnitude of m_x, more likely it is that the final state will solely depend on the sign of m_x as shown in figure S5 (c) and (d).

S4: Theorems to prove that the MTJ based systems functionality is equivalent to that in equation (S1-S2)

As explained in the main article, the state of each MTJ can be measured as a voltage and the input to each MTJ is supplied as a current through the HM layer. However, for simplicity, we will not discuss this resistance to voltage conversions in this section. We will elaborate only using the magnetization dynamics along the easy axis of the MTJs since it gives a direct mapping to the resistance of the MTJs, and thus the output voltage. There are two types of MTJs in our design; a type and s type. The states (resistances) of s type MTJs ($s_i = [s_{i,x} \quad s_{i,y} \quad s_{i,z}]^T, \forall i \leq N$) give the solution to a satisfiability problem $\mathcal{F}$ and the states of a type MTJs ($a_m = [a_{m,x} \quad a_{m,y} \quad a_{m,z}]^T, \forall m \leq M$) exhibit the "satisfiedness" of the respective clauses. The easy axis of both a and s type MTJs is the $\hat{x}$ axis and the pinned layer magnetization is pointed towards $-\hat{x}$ direction. We define the FL magnetization

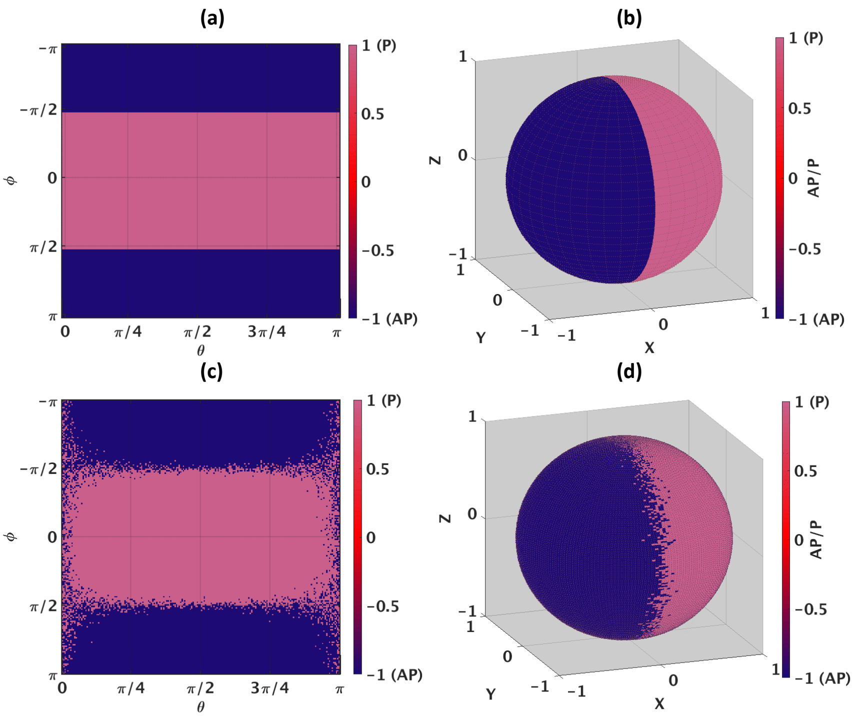

Figure S5: The color shows the state to which the free layer magnetization settles down in our proposed IMA-MTJ device, when there is zero current or external field applied on the MTJ, with an initial condition (ϕ,θ). (a), (b) show the converged final state in absence of thermal noise. (c), (d) show the converged final state when noise is present. (b) and (d) show the same final states in (a) and (c) respectively, in 3 dimensional space.

component along the easy axis of s type MTJs as $-1 \leq s_{i,x} \leq 1$. When the system has converged to a solution, $s_{i,x} = +1$ ($s_{i,x} = -1$) represents $x_i = 1$ ($x_i = 0$) in the Boolean formula $\mathcal{F}$ for $\forall i \leq N$. For the a type MTJs, the FL magnetization component along the easy axis is $a_{m,x}$. When the system has converged to a solution, clause m in $\mathcal{F}$ must be satisfied, and $a_{m,x} = -1$ for $\forall m \leq M$. Further, we apply a rectifying function similar to $g()$ on $a_{m,x}$. The direction of current is defined such that, a positive (negative) current will drive that MTJ towards anti parallel (parallel) state. We will not use the effect of noise and process variations for the proof of the following theorems. Such effects will be analyzed separately as mentioned previously.

$$\left(\frac{1+\alpha^2}{|\gamma|}\right)\frac{\partial s_{i,x}}{\partial t} = \alpha s_{i,x} M_s (1 - s_{i,x}^2)(N_{yy} - N_{xx}) + k_{s,STT}(1 - s_{i,x}^2)\sum_m c_{mi}\, g(a_{m,x}) \tag{S13}$$

$$\left(\frac{1+\alpha^2}{|\gamma|}\right)\frac{\partial a_{m,x}}{\partial t} = \alpha a_{m,x} M_s (1 - a_{m,x}^2)(N_{yy} - N_{xx}) + k_{a,STT}(1 - a_{m,x}^2)(1 - k - \sum_i c_{mi} s_{i,x}) \tag{S14}$$

$$k_{s,STT} = \frac{\hbar k_{she,s} I_s \epsilon}{2q\mu_0 M_s V_s}, k_{a,STT} = \frac{\hbar k_{she,a} I_a \epsilon}{2q\mu_0 M_s V_a}$$

$$k_{she,s} = \frac{A_{MTJ,s}\theta_{she}}{A_{HM,s}}\left(1 - sech\left(\frac{t_{HM,s}}{\lambda_{sf}}\right)\right), k_{she,a} = \frac{A_{MTJ,a}\theta_{she}}{A_{HM,a}}\left(1 - sech\left(\frac{t_{HM,a}}{\lambda_{sf}}\right)\right)$$

$$c_{mi} = \begin{cases} 1 & if\ x_i \in C_m \\ -1 & if\ \overline{x}_i \in C_m \\ 0 & if\ x_i \notin C_m\ and\ \overline{x}_i \notin C_m \end{cases}$$

The above equation set defines the behavior of our MTJ based SAT solver. The subscripts s and a denotes that the given parameter is related to the MTJs that represent variable s and a, respectively. M_s is the saturation magnetization, I is the charge current through the HM layer, V is the volume of the FL, θ_{she} is the Spin Hall Effect (SHE) angle, λ_{sf} is the spin flip length, t_{HM} is the thickness of the FL, A_{MTJ} is the area of the FL at the HM and FL interface, and A_{HM} is the area perpendicular to the charge current through the HM. C_m is the mth clause. Note that including the field-like torque term (equation S7 must be updated in this case to $\overrightarrow{STT} = |\gamma|\beta\Big(\widehat{m} \times \big(\epsilon\widehat{m} \times \widehat{p} + \epsilon^{'}\widehat{p}\big)\Big)$, where ϵ and $\epsilon^{'}$ are dimensionless factors that describe the effectiveness of the spin transfer process) changes the above $k_{s,STT}$ and $k_{a,STT}$ terms (equation S13, S14). Instead of ϵ in these terms, a new factor of $\epsilon + \alpha\epsilon^{'}$ will be present. This can be viewed as a slight increment to the charge current (I_s, I_a). However, due to the small damping factor α, this effect is smaller and thus not considered for the following analysis.

Theorem 1 : Variables s and a remain bounded

$s_i = [s_{i,x} \quad s_{i,y} \quad s_{i,z}]$ and $a_m = [a_{m,x} \quad a_{m,y} \quad a_{m,z}]$ are unit magnetization vectors. $s_{i,x}$ and $a_{m,x}$ are the components along the easy axis $\hat{x}$, as defined earlier. The highest possible value of these are $+1$ and the lowest is -1.

Therefore all $s_{i,x}$ and $a_{m,x}$ remain bounded for all time instances.

Theorem 2: Every k-SAT solution has a corresponding stable fixed point

Let $s^*_{i,x}$ be a solution to a SAT formula $\mathcal{F}$ for $i = 1, 2, ..., N$. The point (s^*_x, a^*_x) where

$$s^*_{i,x} = \pm 1, \forall i \leq N \quad a^*_{m,x} = -1, \forall m \leq M$$

is a stable fixed point of the dynamic system.

According to the equations S13 and S14, it is evident that both $\frac{\partial s^*_{i,x}}{\partial t}$ and $\frac{\partial a^*_{m,x}}{\partial t}$ are zero. (note that $a^*_{m,x} < 0, g(a^*_{m,x}) = 0$). Therefore the point (s^*_x, a^*_x) is a fixed point. In order to prove stability, we shall give a small perturbation of $|s^*_{i,x}| - |s_{i,x}| = \delta_{i,s}$ and $a_{m,x} - a^*_{m,x} = \delta_{m,a}$. Note that $|s_{i,x}| \leq 1$ and $|a_{m,x}| \leq 1$ since they are components of unit vectors s_i and a_m. Such perturbations will result in positive $(1 - s^2_{i,x}\ )$ and positive $(1 - a^2_{m,x})$. If the variable x_i is present in the mth clause ($c_{mi} \neq 0$), and if the clause is satisfied by $s^*_{i,x}$, then $c_{mi}s^*_{i,x} = 1$. If the clause is not satisfied, then $c_{mi}s^*_{i,x} = -1$. Accordingly the sum $\sum_i c_{mi}s^*_{i,x}$ can take $k+1$ possible values; $-k$, $-k+2$,...,$k-2$, k. The value $-k$ corresponds to the mth clause not being satisfied. Other cases imply that there exists at least one variable satisfying the clause. As per our assumption that $s^*_{i,x}$ is a solution of the SAT problem $\mathcal{F}$,

$$\sum_i c_{mi}s^*_{i,x} \geq -k + 2 \quad \rightarrow \quad 1 - k - \sum_i c_{mi}s^*_{i,x} \leq -1$$

Due to the perturbation, there will be a negative current through HM in the a type MTJs. This will shift $a_{m,x}$ towards $a^*_{m,x}$ (*i.e.* towards -1 or parallel state). As long as $|\delta_{m,a}| < 1$, the $a_{m,x}$ after thresholding $g(a_{m,x})$ is zero, leading to a zero current through the HM of the s type devices. When there is no current through the HM of the s type devices, as long as $|\delta_{i,s}| < 1$, $s_{i,x}$ will reach $s^*_{i,x}$. Therefore, the solution point (s^*_x, a^*_x) is a stable fixed point.

Theorem 3 : A stable fixed point always corresponds to a solution

Let us assume that there exists a stable fixed point (s^*_x, a^*_x) for the Boolean SAT problem with some unsatisfied clauses where $|s^*_{i,x}| = 1$ and $|a^*_{m,x}| = 1$. Since $1 - {s^*_{i,x}}^2 = 0$ and $1 - {a^*_{m,x}}^2 = 0$, automatically

$$\Big(\frac{1+\alpha^2}{|\gamma|}\Big)\frac{\partial s^*_{i,x}}{\partial t} = 0, \quad \Big(\frac{1+\alpha^2}{|\gamma|}\Big)\frac{\partial a^*_{m,x}}{\partial t} = 0$$

By multiplying both sides of the equation S13 by $sign(s^*_{i,x})$, we obtain

$$\Big(\frac{1+\alpha^2}{|\gamma|}\Big)\frac{\partial|s^*_{i,x}|}{\partial t} = \alpha|s^*_{i,x}|M_s(1-{s^*_{i,x}}^2)(N_{yy}-N_{xx}) + k_{s,STT}(1-{s^*_{i,x}}^2)\sum_m sign(s^*_{i,x})c_{mi}g(a_{m,x})$$

since (s^*_x, a^*_x) is a fixed point by assumption,

$$(1-{s^*_{i,x}}^2)\left(\alpha|s^*_{i,x}|M_s(N_{yy}-N_{xx}) + k_{s,STT}\Big(\sum_{sign(s^*_{i,x})c_{mi}=1} g(a^*_{m,x}) - \sum_{sign(s^*_{i,x})c_{ni}=-1} g(a^*_{n,x})\Big)\right)$$
$$= 0$$

Let us assume that there are P number of unsatisfied clauses in which the variable x_i or $\bar{x}_i$ appears.

$$\sum_{sign(s^*_{i,x})c_{ni}=-1} g(a^*_{n,x}) = P$$

For the other clauses that are satisfied at the fixed point, $g(a^*_{m,x}) = 0$. This will lead to,

$$(1-{s^*_{i,x}}^2)\Big(\alpha|s^*_{i,x}|M_s(N_{yy}-N_{xx}) - k_{s,STT}P\Big) = 0$$

However, since $\frac{k_{s,STT}}{\alpha M_s(N_{yy}-N_{xx})} > 1$ depending upon the typical values of parameters, $|s^*_{i,x}| \neq \frac{k_{s,STT}P}{\alpha M_s(N_{yy}-N_{xx})}$. This leads to $(1-{s^*_{i,x}}^2) = 0$ become the sole reason for the existence of the stationary point. Similar situation exists for state dynamics of a-type MTJs. Let us consider an unsatisfied clause m, and the corresponding dynamics. As mentioned previously, for an unsatisfied clause, $\sum_i c_{mi}s^*_{i,x} = -k$. Further, since $a^*_{m,x}$ is a stationary point as per our assumption, $\Big(\frac{1+\alpha^2}{|\gamma|}\Big)\frac{\partial a^*_{m,x}}{\partial t} = 0$. Therefore

$$(1-{a^*_{m,x}}^2)\Big(\alpha a^*_{m,x}M_s(N_{yy}-N_{xx}) + k_{a,STT}\Big) = 0$$

However, $a^*_{m,x} \neq -\frac{k_{a,STT}}{\alpha M_s(N_{yy}-N_{xx})}$ since it violates our initial assumption that the clause is not satisfied ($a^*_{m,x} > 0$). Note that $N_{yy} > N_{xx}$. This leads to $(1-{a^*_{m,x}}^2) = 0$ become the sole reason for the existence of the stationary point. Now let us introduce small perturbations $\delta_{i,s}$ and $\delta_{m,a}$ to the system such that, $|s^*_{i,x}| - |s_{i,x}| = \delta_{i,s}$ and $a_{m,x} - a^*_{m,x} = \delta_{m,a}$. As mentioned in theorem 2, this will lead to

$$(1-{s_{i,x}}^2) > 0, \quad (1-{a_{m,x}}^2) > 0$$

Since the perturbation is small, $|s_{i,x}| \cong 1, |a_{m,x}| \cong 1$ is valid as well. When $s_{i,x} \cong +1$, only the clauses where the variable appears as its complement ($c_{mi} = -1$) are not satisfied. Similarly, when $s_{i,x} \cong -1$, the clauses where $c_{mi} = 1$ are not satisfied.

$$\Big(\frac{1+\alpha^2}{|\gamma|}\Big)\frac{\partial s_{i,x}}{\partial t} = \begin{cases}(1-{s_{i,x}}^2)\Big(\alpha s_{i,x}M_s(N_{yy}-N_{xx}) - k_{s,STT}P_-\Big) < 0, for s_{i,x} > 0\\ (1-{s_{i,x}}^2)\Big(\alpha s_{i,x}M_s(N_{yy}-N_{xx}) + k_{s,STT}P_+\Big) > 0, for s_{i,x} < 0\end{cases}$$

Where $P_\pm = \sum_{c_{mi}=\pm1} g(a_{m,x})$. In the light of this, it can be observed that there exists an unstable direction along which the dynamics can escape from the stationary point (s^*_x, a^*_x). That is, $||s^*_{i,x} - s_{i,x}(t)|| \leq \delta$ for any $t > t_0$ is not valid (with t_0 being the time at which the perturbation is applied). Therefore, this disproves the initial assumption that (s^*_x, a^*_x) is a stable stationary point. Therefore, if there exists a stable stationary point in the defined continuous system, they correspond only to solutions of the SAT problem $\mathcal{F}$.

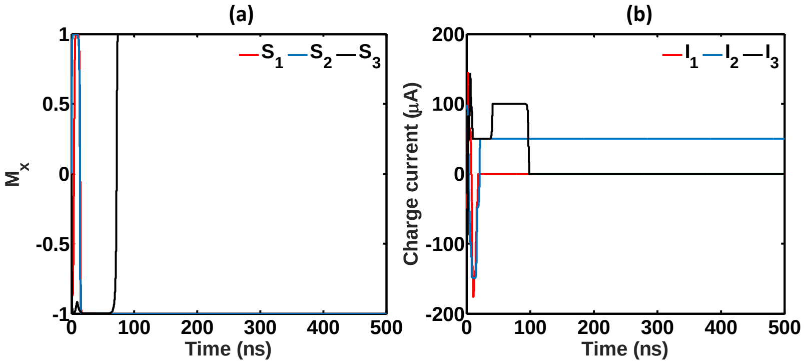

Figure S6: The effect of absence of thermal noise on the operation of the SAT solver. (a) The states of 3 MTJs that represent three s variables (S_1, S_2, S_3) when the thermal noise is not present. The system seems as if it has stabilized even though the solution is not correct. (b) The charge current through the same three MTJs in (a). A positive current drives an MTJ towards the AP state (+1) and a negative current will drive it towards the P state (−1). Note that the positive current through the second device is insufficient to flip the state

S5: Frozen non-solution states under zero thermal noise

The existence of noise is mandatory for the proper operation of our MTJ based SAT solver. As implied by equation (S13) and (S14) in section S4, the state $\hat{m} = [m_x\hat{x} \quad m_y\hat{y} \quad m_z\hat{z}] = [\pm 1 \quad 0 \quad 0]$ of a magnet is stable in the absence of noise. Any magnitude of current (other than ∞) is not capable of switching the magnet as long as the magnetization is perfectly aligned with the positive or negative $\hat{x}$ direction (easy axis). In our proposed system, the switching between the two stable states of a set of MTJs should continuously occur until the system converges to a solution. However, when the thermal noise is not present, a magnetization can end up in the $[m_x\hat{x} \quad m_y\hat{y} \quad m_z\hat{z}] = [\pm 1 \quad 0 \quad 0]$ state. If the state of that MTJ must be changed in the process of converging to a solution, it will be impossible. When thermal noise is present, it will induce a slight tilt in the FL magnetization with the $\hat{x}$ direction. When trying to switch an MTJ using a current, this tilt of the FL magnetization has a significant contribution. The switching time between two states of an MTJ is a strong function of this tilt.[12] When the tilt is zero, the switching time can be explained as infinite. Figure S6 illustrates the time evolution of the states of three MTJs, that represent three variables when solving a 3-SAT problem with 20 variables. The effect of thermal noise is ignored for this case. We have forced an initial condition for the system to $M_x = 0$ (*i.e.* the resistance values of all the MTJs that represent the variable s is $(R_{ap} + R_p)/2$). This can be done by passing a current through the heavy metal, perpendicular to the write current direction in figure 1 (c) (main article). Even though the states show that they have stabilized (note S_1, S_2, and S_3 after 75ns in figure S6(a)), there exists a current through the heavy metal layer of the second device (note I_2 is not zero after 75ns in figure S6(b)). This current tries to drive the state of the MTJ towards the other direction. Note, a positive current drives an MTJ towards the AP state (+1) and a negative current will drive it towards the P state (−1). Despite the presence of current (I_2) through the second device, the magnet does not flip to $[+1 \quad 0 \quad 0]$ state. This is because the tilt the FL magnetization has with the easy axis is too small, and the current is not sufficient to drive the state towards $[+1 \quad 0 \quad 0]$. This is a 'frozen non-solution state' that occurs only in the absence of thermal noise. Therefore, it is evident that the system fails under zero thermal noise.

S6: Effects of process variations

During the fabrication process, non-idealities such as edge damage[13,14] can be introduced to the MTJs. Such non-idealities can change the parameters associated with the devices and might affect the performance of the proposed SAT solver. We invesitigate the effect of global variations of three parameters, *viz.* interface anisotropy energy density constant (K_i), width and length.

The changes in K_i due to edge damage can be modeled as a linearly varying K_i over a small length from the edge towards the center of the MTJ.[13] For the experiments, we assumed that the K_i

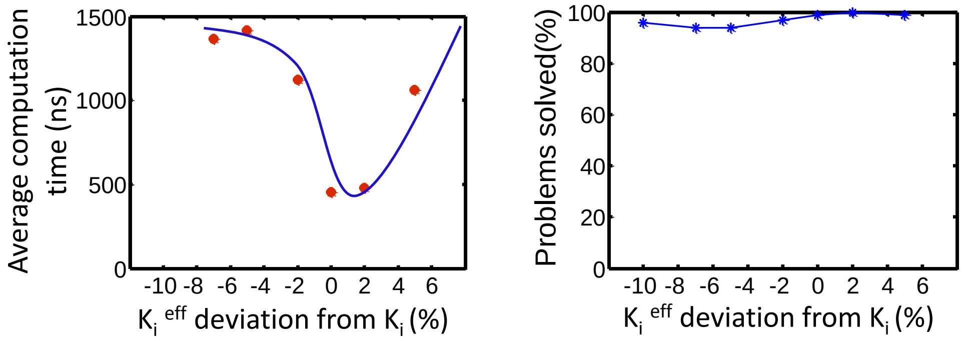

Figure S7: The effect of global variations in interface anisotropy energy density constant K_i, over the (a) computation time of the system, and (b) the percentage of 20-variable SAT instances solved within 10μs

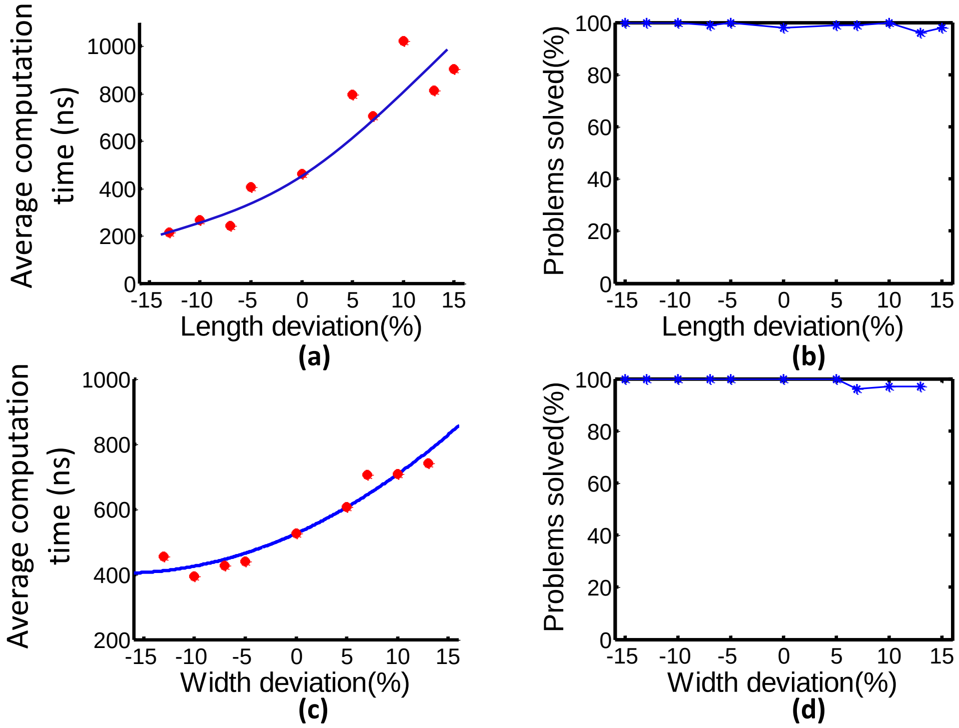

Figure S8: The effect of global variations in lengths and widths of MTJs over the computation time of the system, and the percentage of 20-variable SAT instances solved within 10μs. (a),(b) shows the effects on computation time and percentage of problems solved when the length is varied from -15% to $+15\%$, with respect to the nominal length. (c) and (d) respectively show the effects on computation time and percentage of problems solved, when the width of the free layer is varied

variations near the edge can be represented by an effective K_i (K_i^{eff}) throughout the magnet. Figure S7 shows how the computation time changes due to global variations in the K_i^{eff} as a percentage from the nominal K_i.

We observed that the increased/decreased K_i^{eff} results in increased computation time. This is due to the fact that t_{ss} is dependent upon K_i (equation S11). Since increased K_i^{eff} results in increased t_{ss} and vice versa, the trend in figure S7 is similar to the mirror image (along y axis) of the 'computation time vs thickness' curve in figure 9 (b) in the main article. However, figure S7 (b) shows that the percentage of problems solved does not have a significant impact from the K_i^{eff} variations.

We further observed how the changes in shape anisotropy (due to changes in dimensions) affect

the performance of our MTJ based SAT solver. We induced global variations in the range of $\pm 15\%$ to the widths and lengths of the MTJs and observed the change in computation time by means of 20-variable hard SAT instances. Figure S8 summarizes the results obtained. Figure S8 (a) and (c) illustrate that, decreased dimensions decreases the computation time and vice versa. This is due to the reduction in the energy barrier with reduced dimensions. We thus conclude that the reduced dimensions improves the performance of our solver.

Intuitively, since the non-ideal effects such as edge damage can potentially reduce the switching current of a magnet,[13] the computation time of our system must reduce. If an edge damage free-MTJ switches within t_1 amount of time due to an I_1 current, a magnet with edge damage switches faster than t_1 for the same current, I_1. To view the above effect on computation time of our solver, we increased the applied current on all MTJs by 10%. This resulted in an average computation time reduction of about 36% for 20-variable hard SAT problems.

As explained in prior work,[13] the effects of edge damage is minimal on MTJs with larger dimensions. Furthermore, it has been shown[14] that using smaller accelerating voltages during the ion milling fabrication process reduces the edge damage as well. These counter measures can be exploited to avoid consequent performance degradation of the proposed SAT solver if required.

References

[1] B. Molnár and M. Ercsey-Ravasz, "Asymmetric continuous-time neural networks without local traps for solving constraint satisfaction problems," *PloS one*, vol. 8, no. 9, p. e73400, 2013.

[2] S. Kirkpatrick, B. Selman, *et al.*, "Critical behavior in the satisfiability of random boolean expressions," *Science-AAAS-Weekly Paper Edition-including Guide to Scientific Information*, vol. 264, no. 5163, pp. 1297–1300, 1994.

[3] F. Krzakała, A. Montanari, F. Ricci-Tersenghi, G. Semerjian, and L. Zdeborová, "Gibbs states and the set of solutions of random constraint satisfaction problems," *Proceedings of the National Academy of Sciences*, vol. 104, no. 25, pp. 10318–10323, 2007.

[4] F. Krzakała and L. Zdeborová, "Phase transitions and computational difficulty in random constraint satisfaction problems," in *Journal of Physics: Conference Series*, vol. 95, p. 012012, IOP Publishing, 2008.

[5] S. A. Cook, "The complexity of theorem-proving procedures," in *Proceedings of the third annual ACM symposium on Theory of computing*, pp. 151–158, ACM, 1971.

[6] J. C. Slonczewski, "Conductance and exchange coupling of two ferromagnets separated by a tunneling barrier," *Physical Review B*, vol. 39, no. 10, p. 6995, 1989.

[7] C.-F. Pai *et al.*, "Spin transfer torque devices utilizing the giant spin hall effect of tungsten," *Applied Physics Letters*, vol. 101, no. 12, p. 122404, 2012.

[8] S. Ikeda *et al.*, "A perpendicular-anisotropy cofeb–mgo magnetic tunnel junction," *Nature materials*, vol. 9, no. 9, pp. 721–724, 2010.

[9] Z. Wang, G. Yu, X. Liu, B. Zhang, X. Chen, and W. Lu, "Magnetization characteristic of ferromagnetic thin strip by measuring anisotropic magnetoresistance and ferromagnetic resonance," *Solid State Communications*, vol. 182, pp. 10–13, 2014.

[10] A. Jaiswal and K. Roy, "Mesl: Proposal for a non-volatile cascadable magneto-electric spin logic," *Scientific reports*, vol. 7, 2017.

[11] A. Aharoni, "Demagnetizing factors for rectangular ferromagnetic prisms," *Journal of applied physics*, vol. 83, no. 6, pp. 3432–3434, 1998.

[12] Y. Huai, "Spin-transfer torque mram (stt-mram): Challenges and prospects," *AAPPS bulletin*, vol. 18, no. 6, pp. 33–40, 2008.

[13] K. Song and K.-J. Lee, "Spin-transfer-torque efficiency enhanced by edge-damage of perpendicular magnetic random access memories," *Journal of Applied Physics*, vol. 118, no. 5, p. 053912, 2015.

[14] Z. Sun, S. Retterer, and D. Li, "The influence of ion-milling damage to magnetic properties of co80pt20 patterned perpendicular media," *Journal of Physics D: Applied Physics*, vol. 47, no. 10, p. 105001, 2014.
